# Field-Grown and In Vitro Propagated Round-Leaved Sundew (*Drosera rotundifolia* L.) Show Differences in Metabolic Profiles and Biological Activities

**DOI:** 10.3390/molecules26123581

**Published:** 2021-06-11

**Authors:** Jenni Tienaho, Dhanik Reshamwala, Maarit Karonen, Niko Silvan, Leila Korpela, Varpu Marjomäki, Tytti Sarjala

**Affiliations:** 1 Biomass Characterization and Properties Group, Production Systems Unit, Natural Resources Institute Finland, Latokartanonkaari 9, FI-00791 Helsinki, Finland; niko.silvan@luke.fi (N.S.); tytti.sarjala@luke.fi (T.S.); 2 Department of Biological and Environmental Science, University of Jyväskylä, Seminaarinkatu 15, FI-40014 Jyväskylä, Finland; dhanik.d.reshamwala@jyu.fi (D.R.); varpu.s.marjomaki@jyu.fi (V.M.); 3 Natural Chemistry Research Group, University of Turku, FI-20014 Turku, Finland; maarit.karonen@utu.fi; 4 Forest Health and Biodiversity Group, Natural Resources Unit, Natural Resources Institute Finland, FI-00791 Helsinki, Finland; leila.korpela@luke.fi

**Keywords:** *Drosera rotundifolia*, antioxidants, antiviral properties, phenolic compounds, secondary metabolites

## Abstract

*Drosera rotundifolia* L. is a carnivorous plant used in traditional medicine for its therapeutic properties. Because of its small size, its collection in nature is laborious and different cultivation methods have been studied to ensure availability. However, only a few studies exist where the lab-grown sundew tissue and field-grown sundew would have been compared in their functionality or metabolic profiles. In this study, the antioxidant and antiviral activities of lab-grown and field-grown sundew extracts and their metabolic profiles are examined. The effect of drying methods on the chromatographic profile of the extracts is also shown. Antioxidant activity was significantly higher (5–6 times) in field-grown sundew but antiviral activity against enterovirus strains coxsackievirus A9 and B3 was similar in higher extract concentrations (cell viability ca. 90%). Metabolic profiles showed that the majority of the identified compounds were the same but field-grown sundew contained higher numbers and amounts of secondary metabolites. Freeze-drying, herbal dryer, and oven or room temperature drying of the extract significantly decreased the metabolite content from −72% up to −100%. Freezing was the best option to preserve the metabolic composition of the sundew extract. In conclusion, when accurately handled, the lab-grown sundew possesses promising antiviral properties, but the secondary metabolite content needs to be higher for it to be considered as a good alternative for the field-grown sundew.

## 1. Introduction

Round-leaved sundews (*Drosera rotundifolia* L.) are small carnivorous plants growing in Northern peatlands and other nutrient-poor soils [1,2]. They secrete sugary glowing mucilage into the hairs covering their leaves and feed on the attracted insects, which get caught on the sticky liquid and are then enzymatically dissolved [1,3]. *D. rotundifolia* is used in traditional medicine, where its ability to relieve coughs and pulmonary diseases has been acknowledged for centuries [2,3,4]. The therapeutic activities have mainly been associated with two compound groups: flavonoids and naphthoquinones. For example, the anti-inflammatory [5] and antimicrobial [6,7,8] activities of *D. rotundifolia* have been established and the drug Droserae Herba, which is traditionally produced from the dried aerial parts of the plant, is accepted in various pharmacopeias of the world [3,4]. Some naphthoquinones and flavonoids, which are important secondary metabolites of *Drosera* have also been considered antiviral [9,10]. In addition, *Drosera* has in some cases been identified with antioxidant activity and iron-chelating properties [11]. The secondary metabolites of *Drosera* can for example be of use in the pharmaceutical, cosmetics, and food industry [12]. The round-leaved sundew is not endangered in Finland [13], but the collection of small-sized plants from natural stands is laborious and therefore cultivation methods [2,14] and in vitro propagation [2,12,15] have been studied to achieve better availability of the valuable plant material. The chemical composition and functional properties may vary in sundew plants depending on the growth conditions and site [16]. Therefore, the potential differences in the properties of field-grown and in vitro propagated plant material should be studied. In an earlier study, we demonstrated that antibacterial activity was significantly higher in field-grown *D. rotundifolia* ethanol extract in comparison to the propagated sundew extracts [8]. However, only a few studies exist where the functional properties and metabolic profiles of propagated and field-grown sundews are compared.

In this study, we investigate and compare the antioxidant (oxygen radical absorbance capacity (ORAC), ferric reducing antioxidant power (FRAP), H_2_O_2_ scavenging) and antiviral (enteroviruses CVA9 and CVB3) activities of both propagated and field-grown sundew, and describe the differences in their metabolic profiles using UPLC-DAD-ESI-QOrbitrap-MS/MS. We also describe the effects of different handling techniques, such as the drying of the extracts, on their chromatographic profile.

## 2. Results and Discussion

### 2.1. Growth of the Laboratory Propagated Sundew Tissue

**Figure 1:**
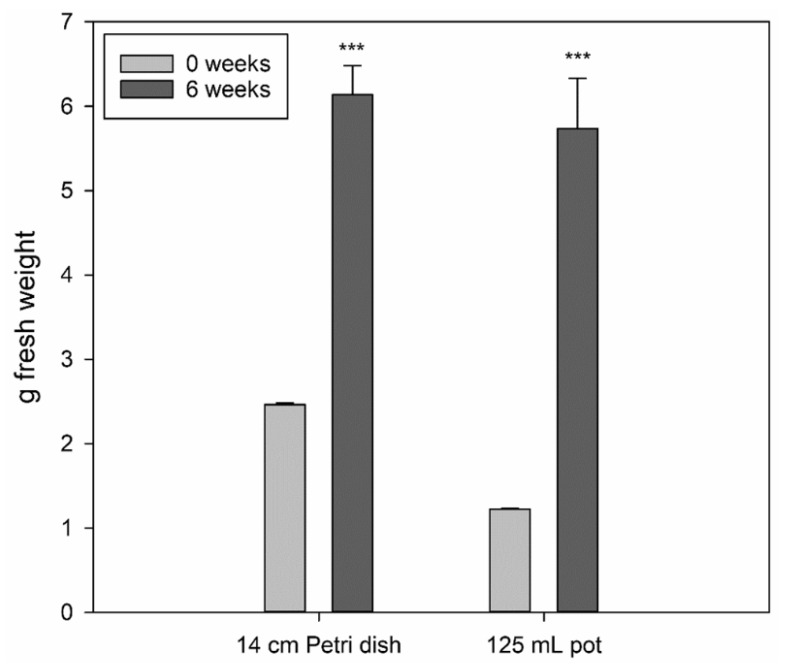
Yield (g fresh weight, mean ± stdev, n = 6) of the total propagated sundew tissue in six weeks on ½ MS medium on Petri dishes and 125 mL pots. A one-way ANOVA test was used to assess the statistical significance of differences between 0 weeks and 6 weeks (*** = *p* < 0.001). No significant difference was observed between Petri dish and pot-grown biomass after 6 weeks.

**Figure 2:**
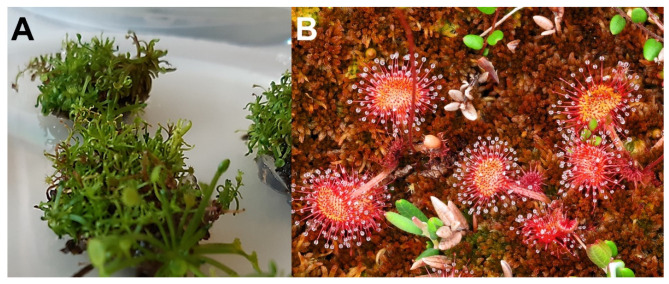
The sundew plants used in this study: (**A**) The in vitro propagation of *D. rotundifolia* on a Petri dish (photo: Tytti Sarjala) and (**B**) sundew growing wild on *Sphagnum* moss (photo: Hannu Nousiainen). The sundew cultivations in pots were morphologically alike to the tissue in figure (**A**).

The laboratory propagated sundew tissues grew well on the ½ MS medium. The fresh weight of the tissues increased 250% in six weeks when growing on Petri dishes (ᴓ 14 cm) and 470% when growing in 125 mL pots (Figure 1). No significant difference was observed between the final biomass levels from the pots or Petri dishes (*p*-value = 0.605), whereas significance was established between the initial and final biomass levels of the pots and Petri dishes (both with *p*-values < 0.001). This suggests that by selecting the growth conditions it is possible to optimize and significantly improve the yield of sundew tissue production. The morphological features of the propagated tissue showed the development of green leaf structures without red color or a proper stem (Figure 2A), thus lacking the typical look of sundew plants grown in a natural environment (Figure 2B).

The growth potential of propagated sundew tissue was promising, showing an increase of over 400% in fresh weight within six weeks. This could probably be further optimized, because only one growth medium was used here.

### 2.2. Antiviral Activity

**Figure 3:**
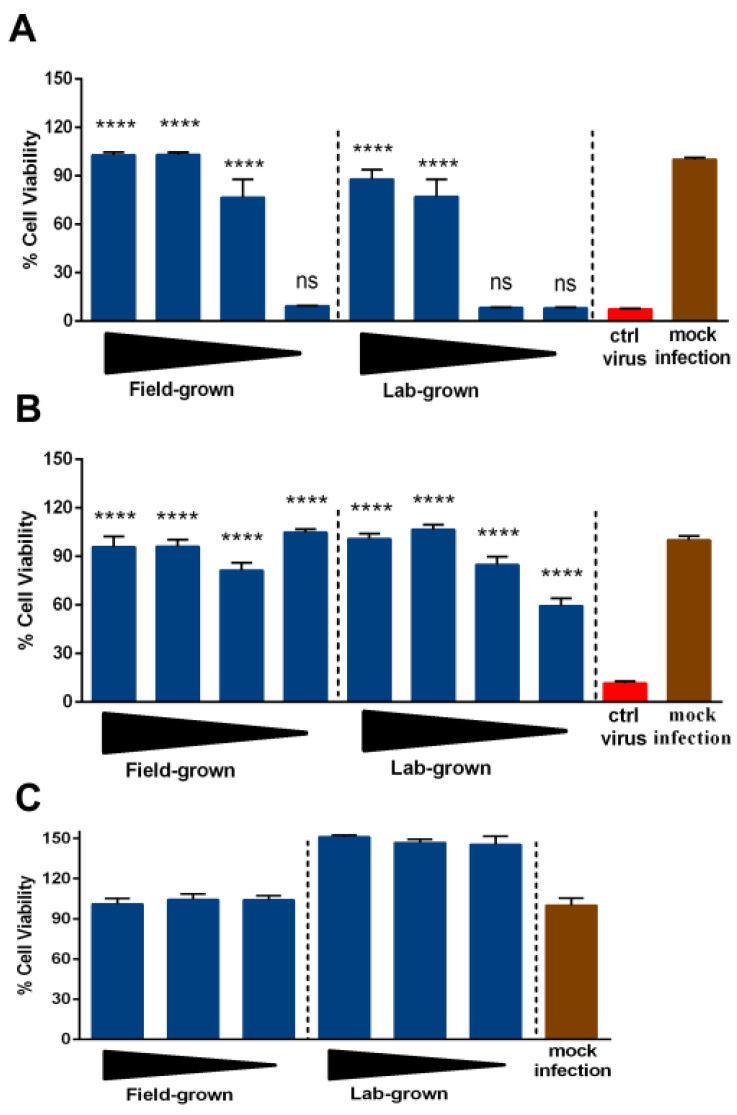
The antiviral efficacy of round-leaved sundew against (**A**) CVA9 and (**B**) CVB3 and its (**C**) cytotoxicity at decreasing concentrations (10%, 5%, and 1% *v/v*) tested on human A549 cells. For the antiviral assessment, CVA9 (2 × 10^6^ PFU/mL) and CVB3 (2 × 10^5^ PFU/mL) were treated with decreasing concentrations (10%, 5%, 1%, and 0.1%, *v/v*) of the round-leaved sundew. Average value + standard errors of mean (SEM) are shown. One-way ANOVA followed by Bonferroni test were used to assess the statistical significance of differences between the virus control and test samples (**** = *p* < 0.0001; ns = non-significant).

The antiviral potential of the round-leaved sundew was determined against non-enveloped enteroviruses CVB3 and CVA9 in our assays. Enteroviruses are responsible for numerous acute and chronic infections globally [17,18]. Compared with enveloped viruses, these non-enveloped viruses are quite stable and resistant to disinfectants [19]. Chemical-based disinfectants have other limitations in terms of their toxicity and being hazardous to health. Here, we tested various concentrations (10%, 5%, 1%, and 0.1%, *v/v*) of field-grown and in vitro propagated/lab-grown sundew to assess their ability to rescue A549 cells from CVA9 and CVB3 infections. Interestingly, both the field and lab-grown round-leaved sundews were able to protect cells from both virus serotypes. The extracts were more effective against CVA9 compared with CVB3 (Figure 3A,B). In addition, the field-grown sundews showed antiviral activity even at lower concentrations, suggesting that it was better at reducing virus infectivity compared with the lab-grown sundews. It is likely that this phenomenon is affected by the metabolic profile of the extracts. For example, Lin et al. [20] found that polyphenols, such as tannins are effective against coxsackieviruses. None of the concentrations tested were cytotoxic (Figure 3C), which was evaluated using CPE Inhibition assay. Based on our findings, the extracts are acting directly on the virus capsid as they showed good antiviral activity when the virus was pre-treated with them for 1 h at +37 °C. In the future, it would be interesting to study, the actual antiviral mechanism of these extracts.

### 2.3. Antioxidant Activity

**Figure 4:**
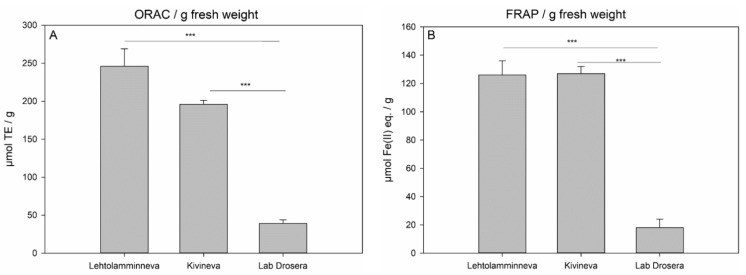
(**A**) ORAC and (**B**) FRAP activities (mean ± standard deviation, *n* = 3) of *D. rotundifolia* plants collected from Lehtolamminneva mire and Kivineva mire and lab-grown propagated *D. rotundifolia* biomass (Lab Drosera). TE = Trolox (vitamin E derivative) equivalent. One-way ANOVA test was used to assess the statistical significance of differences between Lab Drosera and mire grown samples (*** = *p* < 0.001).

Both ORAC and FRAP assays showed that field-grown sundew plants possessed much higher antioxidant properties in comparison with the lab-grown propagated biomass (Figure 4). Significant differences for the ORAC values were observed between the lab *Drosera* and Lehtolamminneva mire sundew (*p*-value < 0.001) and Kivineva mire sundew (*p*-value < 0.001). The difference between the two field-grown sundews was also significant (*p*-value = 0.014). A similar observation was made for the FRAP data, where one-way analysis of variance (ANOVA) gave *p*-values < 0.001 between the lab *Drosera* results and results for the two mires, whereas the difference between Lehtolamminneva mire and Kivineva mire results was insignificant (*p*-value = 0.96). H_2_O_2_ scavenging assay showed very low H_2_O_2_ inhibition % (3.1–3.5%) for all the samples and no differences between the field-grown or lab-grown *Drosera* were found. Changes in the growth conditions (light, nutrients, natural peatland environment) of the propagated tissue did not result in any improvement of the antioxidant properties measured as FRAP. Exposing the propagated sundew tissue for two days to ambient daylight in summer affected the tissue by decreasing FRAP activity by 12% from 18.5 ± 1.0 to 16.3 ± 0.8 µmol Fe(II) eq. per 1 g. A decrease in the nutrient content of the growth medium in comparison with the ½ MS medium at a 30% nutrient level or water squeezed from *Sphagnum* moss also decreased the FRAP activity of the tissue from 42.7 ± 6.8 to 19.1 ± 4.7, 21.9 ± 3.4 µmol Fe(II) eq. per 1 g, respectively. Furthermore, the propagated sundew tissue did not survive in a natural environment on a peatland when transplanted there for two weeks.

### 2.4. Stability under Different Drying Methods

**Figure 5:**
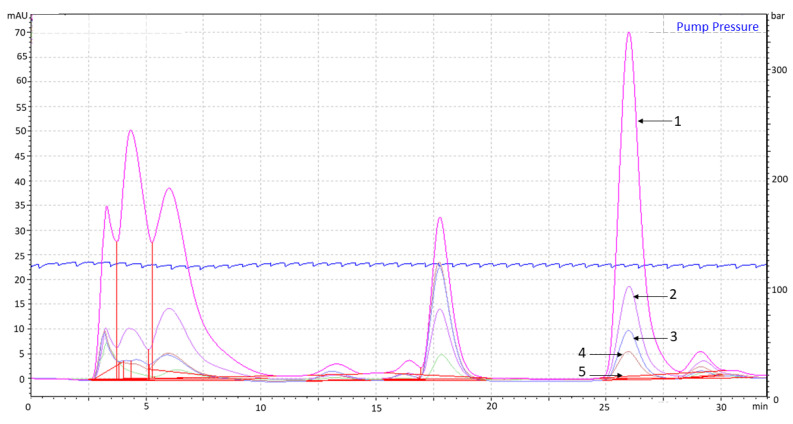
HPLC grams of differently dried sundew tissue extract. The drying treatments of the overlaid HPLC grams are marked as follows: (1) frozen (−20 °C), (2) freeze-drying (43 h), (3) room temperature (43 h), (4) herbal-dryer (+45 °C, 27 h), and (5) oven (+105 °C, 24 h).

HPLC analysis revealed that the compound contents of sundew tissue extract were not stable under drying treatments. The peak area, for example, at a retention time of 26 min in the HPLC gram decreased when compared with the sample of the frozen tissue extract (−20 °C) (100%) to 28%, 15%, 10%, and 0% in the extract of sundew after freeze-drying, room temperature (+20 °C), herbal-dryer (+45 °C), and oven drying (+105 °C), respectively (Figure 5). Identical amounts of the same extract with no variation in the compound content were used for all the handling and drying methods.

### 2.5. Metabolite Profiles

**Figure 6:**
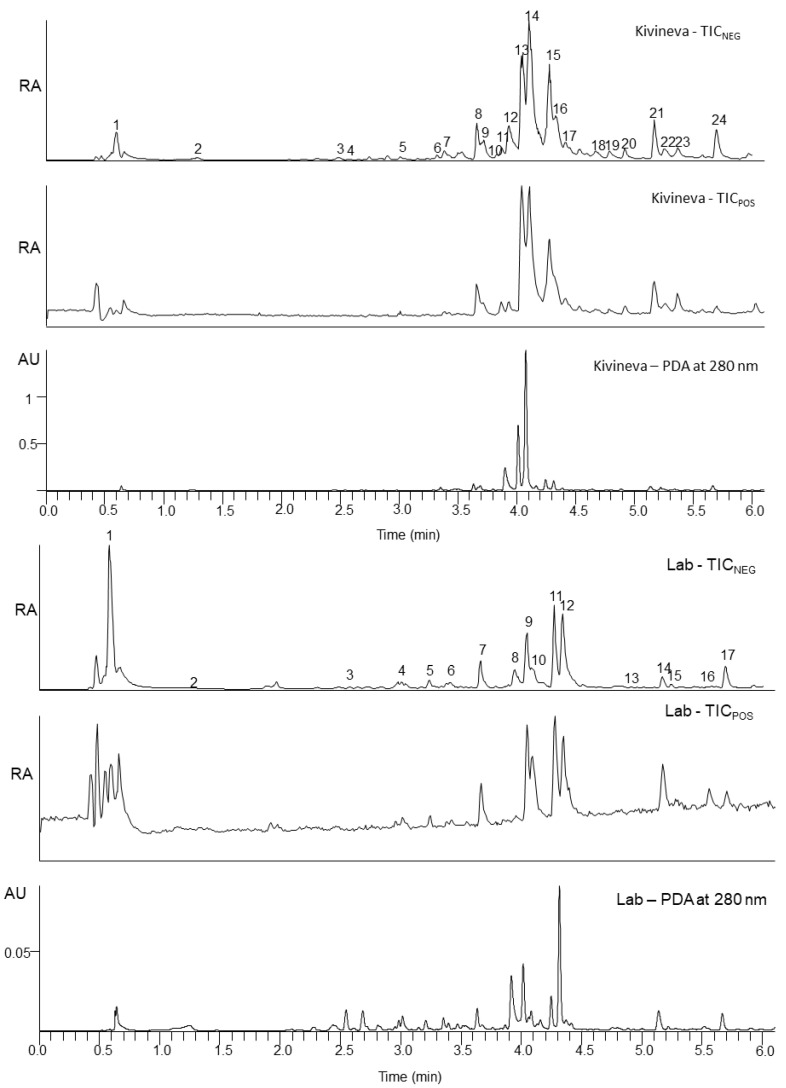
The total ion chromatograms (TIC) in positive and negative ionization and photodiode array (PDA) chromatogram at 280 nm for field-grown sundew (Kivineva mire) on the top and lab-grown propagated sundew (Lab) on the bottom.

The metabolite profiles of field-grown and propagated sundews were investigated from untreated extracts using UPLC-DAD-ESI-QOrbitrap-MS/MS and the results for the field-grown sundews are shown in Table 1 and for lab-grown propagated tissues in Table 2. In addition to the metabolites shown in the tables, saccharides and small organic acids were eluting with very early retention times (RT) in both sundew types (Figure 6). The lab-grown sundew extract also indicated the presence of arginine, which was not found in the field-grown extract. Most of the identified metabolites were similar in the lab and field-grown extracts but also differences were observed. For instance, 14 compounds were found in both extracts, but ten compounds could only be found in the field-grown sundew extract and six in the lab-grown extract. The lab-grown extract contained an isomer of a coumaric acid glycoside eluting at total ion chromatogram (TIC) with a retention time (RT) of 3.04 min, two unknown compounds at RT 3.24 min, hyperoside eluting also at RT 4.09 min, syringetin glycoside eluting at RT 5.24 min, and spinatoside eluting at RT 5.55 min, which were not observed in the field-grown sundew extract. Field-grown sundew extract contained digalloyl glycose at RT 2.49 min, dihydromyricetin at RT 3.32 min, hexahydroxyflavone glycoside at RT 3.72 min, tetrahydroxyflavone at RT 3.83 min, an unknown compound at RT 3.86 min, kaempferol-galloyl-glycoside at RT 4.42 min, quercetin glycoside at RT 4.68 min, methyl ellagic acid at RT 4.79 min, quercetin glycoside gallate at RT 5.25 min, and quercetin at RT 5.37 min. Because the drying methods compromise the metabolic profile and compound stability, the extracts were considerably dilute, which seemed to slightly affect the shape and maxima of UV spectra (Figure 6). Tuominen [21], Zehl et al. [22], Braunberger et al. [23], Jia et al. [24], and Marczak et al. [25] all verify the identification of compounds based on their retention time, elution order, exact masses, and/or characteristic MS/MS fragments.

**Figure 7:**
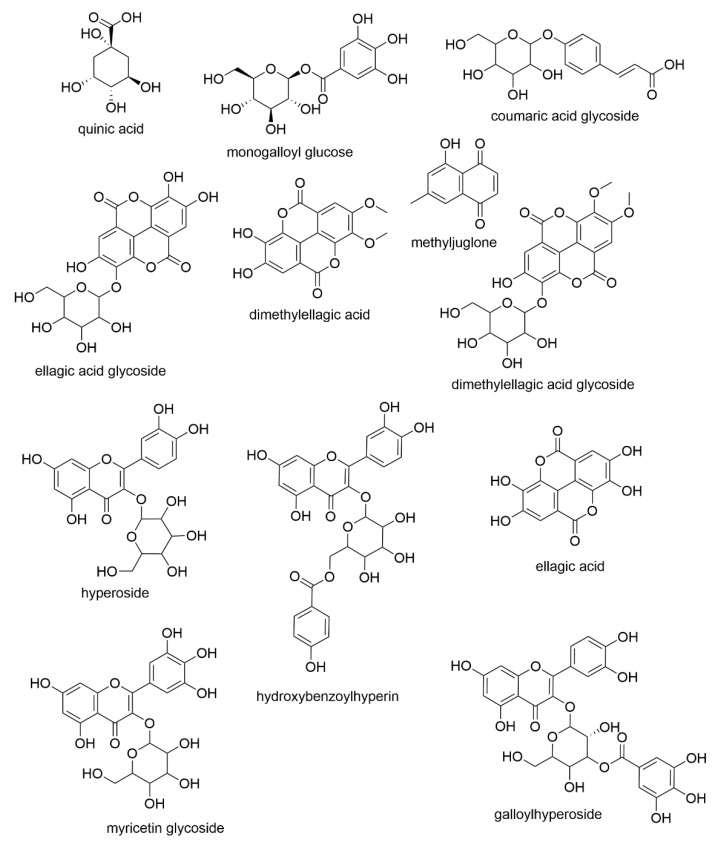
The compounds identified in both field-grown and lab-grown sundew ethanol extracts apart from two methyljuglone glycosides. The positions of the galloyl, benzoyl, and hexose groups are only indicative and could be any free OH-group in the structure.

In Figure 7, 12 compounds found in both extracts are shown. The majority of the compounds are flavonoids, ellagic acid derivatives, or naphthoquinones. Two methyljuglone glycosides are not shown because the methyl group can be attached to the C-7 (7-methyljuglone) or to C-2 (plumbagin).

Quinic acid is a cyclic polyol that has widely been found in plants. Monogalloyl glucose consists of gallic acid and glucose. Hydrolysable tannins (HTs) are a structurally complex group of plant secondary metabolites, which can be divided into three subclasses: simple gallic acid derivatives, gallotannins, and ellagitannins [26]. HTs contain various interesting bioactive properties including antioxidant, antibacterial, antiviral, and anticancer activity [27,28]. As well as gallic acid and ellagic acid, coumaric acid is another phenolic acid and is very common in plants. Phenolic acids have been reported with antioxidant, antimicrobial, and antitumor properties [29]. Flavonoids are an important group of secondary metabolites and plant polyphenols, which are, e.g., responsible for the pigmentation of plants. While flavonoids are not a uniform group, they commonly contain antioxidant, anti-inflammatory, antimicrobial, and antiviral activities [10,30]. Both field-grown and lab-grown extracts contained flavonoids; however, the field-grown sundew was observed to contain significantly higher amounts, which may explain the large difference in the coloration of the field-grown and lab-grown sundew (Figure 2). Of the compounds that were observed from each sundew extract only coumaric acid glycoside and dimethyl ellagic acid glycoside were observed in higher amounts from the lab extract. Naphthoquinones have generally been considered responsible for many of the therapeutic activities in sundew, and they also seem to be more abundant in the field-grown extract. In particular, 7-methyljuglone has been concluded to have antibacterial, antifungal, antiviral, anticancer, and anti-inflammatory properties [31] and it has been observed in Finnish *D. rotundifolia* [16]. While methyljuglone diglycoside has a higher peak area in lab-grown sundew extract, there are two co-eluting compounds, which affect the determined value.

Antioxidants are of use in the food processing industry as preservatives and more recently as active films for packaging and as edible coatings [32]. However, it is important to note that the dosages and possible toxicological effects must be studied in detail before higher consumption of plant materials [32]. Material availability of the field-grown sundew is an issue the needs to be solved before wider use can be considered. The low antioxidant properties of the propagated sundew tissues are related to their chemical characteristics as they contain a lower amount and number of simple phenolics and polyphenols than the field-grown sundews. The modest antioxidant potential of the in vitro grown tissue is a challenge that restrains the potential of further development of the propagation technique unless an appropriate method to improve it is developed. One interesting method to potentially increase the antioxidant activity of the propagated sundew tissue could be a drastic reduction of the mineral content in the growing media. Jadczak et al. [15] found that when the mineral content of the growing media was reduced by 75%, the sundew plants produced the greatest number of long roots, had the highest weight, and were able to produce red coloration in the glandular tentacles. Thus, as the plant would be morphologically more like the field-grown sundew, it could potentially include more phenolic compounds, which are often rich in color and well known for their antioxidant potential. On the other hand, it was fascinating that the propagated sundews showed almost equally high antiviral effects against enterovirus strains CVA9 and CVB3 in high concentrations. With smaller concentrations, the field-grown sundew was more effective, which corresponds with the observation that it contains more effective plant polyphenols. Tannins and other polyphenols have indeed been previously identified with antiviral activities. For example, Lin et al. [20] found that tannins chebulagic acid and punicalagin significantly reduced the coxsackievirus infectivity by both inactivating cell-free viral particles and inhibiting viral binding. In another study by Du et al. [33], resveratrol-loaded nanoparticles were successful in inhibiting enterovirus replication and protecting rhabdosarcoma cells in vitro.

In conclusion, freezing is the best way to maintain the metabolite profile of the sundew extracts, while drying methods compromise the extract profile stability. Antioxidant activity was higher in field-grown extracts and in relation to the secondary metabolite content of the extracts. In higher concentrations, both lab-grown and field-grown extracts are effective against enteroviruses, but the field-grown sundew extract is more effective in smaller concentrations. To increase the antioxidant (this study) and antibacterial [8] activity, the polyphenolic secondary metabolite content of the lab-grown extracts should be increased significantly for it to be considered an effective alternative to the field-grown round-leaved sundew.

## 3. Materials and Methods

### 3.1. Sundew Material, Growth, and Extraction

Sundews grown on peatlands in western Finland and in vitro propagated sundews in a laboratory environment were used in this study. Round-leaved sundew plants (*Drosera rotundifolia*) were collected (26 June 2018) from two peatlands (Lehtolamminneva, 62°6.01′ E 22°57.22′ and Kivineva N 61°57.77′ E 23°23.98′) in Western Finland and stored in a freezer (−20 °C) until extracted and tested.

The in vitro propagated sundew biomass was grown at room temperature (22–25 °C) under continuous fluorescent room light. Seeds of field-grown *D. rotundifolia* were surface sterilized by placing them within folded filter paper and soaking the package first in 70% ethanol for ca. 30 s, followed by 5% sodium hypochlorite for 10–15 min, and rinsing three times in sterile distilled H_2_O. The surface-sterilized seeds were placed on ᴓ 9 cm Petri dishes with a modified half-strength Murashige-Skoog (MS) [34] growing media. The plates contained 2.2 g/L of MS basal medium, 0.039 mg/L FeSO_4_·7H_2_O, 100 mg/L myoinositol, 0.1 mg/L benzyl aminopurine, 0.05 mg/L 1-naphthalene acetic acid, 30 g/L sucrose, and 6.5 g/L of agar. The pH was adjusted to 5.8. Inoculated plates were sealed with parafilm and incubated at room temperature. During the germination, the light cycle was 16 h of light and 8 h of darkness, but, after germination, they were grown under continuous room light (fluorescent lamp). To grow the plant tissue continuously it was repeatedly divided under sterile conditions onto a new ½ MS agar medium without the addition of benzyl aminopurine or 1-naphthalene acetic acid.

For the antioxidant assays, the sundew samples were extracted by grinding 1.0 g fresh weight of plant biomass per 7.0 mL of 99.5% ethanol and incubating the extract in test tubes for one hour by mixing the vials with using a vortex several times during the extraction. The ethanol extract was stored in freeze (−20 °C) until analyzed.

Propagated sundew tissues were weighed before and after transferring to a new medium to define the growth rate under laboratory conditions. The amount of sundew yield at laboratory scale on ᴓ 14 cm Petri dishes and 125 mL pots were compared by starting the growth with 4–8 pieces of 0.3 g sundew tissues and weighing the yield after 6 weeks growth on six Petri dishes and six pots. Furthermore, to study whether natural daylight in summer would affect the antioxidant properties of the propagated sundew tissues, Petri dishes with sundew tissues were moved outside for two days under ambient daylight (day temperature +20 °C), and extracted for the FRAP testing after that. The effect of nutrient availability of the growing medium on the FRAP activity of the propagated sundew tissue was tested by decreasing the nutrient content to 30% of the normal (1/2 MS) and by replacing all the nutrients with soil water squeezed from *Sphagnum* moss collected from the natural peatland site. The propagated tissue was also moved to a natural peatland just beside naturally grown sundew plants for two weeks to monitor the survival in the natural environment.

### 3.2. The Effects of the Drying of the Plant Material on the Properties of the Extract

To elucidate the effect of different drying methods on the properties of sundew plant material the extracts of differently dried sundew tissues were compared with HPLC. Extract of the frozen (–20 °C) sundew tissue was compared with extracts from sundews dried at room temperature (43 h), herbal-dryer (+45 °C, 27 h), freeze-dryer (sample kept frozen, 43 h), or oven dried (+105 °C, 24 h).

### 3.3. Antioxidant Activity

#### 3.3.1. FRAP (Ferric Reducing Antioxidant Power)

Ferric reducing antioxidant power (FRAP) assay is based on single-electron transfer and measures the ability of an antioxidant to reduce ferric (FeIII) to ferrous (FeII) ion [35]. Samples, with three technical replicates in a 96-well format, were used in the assay as described by Vaario et al. [36]. The samples were mixed with 20 mM FeCl_3_·6H_2_O and 10 mM 2,4,6-tris (2-pyridyl)-s-triazine (TPTZ) (both from Sigma-Aldrich Chemie GmbH, Steinheim, Germany) in 300 mM acetate buffer pH 3.6. The absorbance was measured at 594 nm with a fluorescence microplate reader (Varioskan Flash, Thermo Scientific) after the formation of the ferrous-tripyridyltriazine complex in the reaction mixture. FeSO_4_·7H_2_O (Sigma-Aldrich Chemie GmbH, Steinheim, Germany) was used as a standard compound and L(+)-ascorbic acid (150 µM and 800 µM) (VWR Chemicals) as a control and the results are expressed as µmol Fe(II) equivalents per 100 g.

#### 3.3.2. ORAC (Oxygen Radical Absorbance Capacity)

Oxygen Radical Absorbance Capacity (ORAC) assay is based on hydrogen atom transfer and measures the reduction in fluorescence signal caused by the oxidative dissociation of fluorescein in the presence of peroxyl radicals (R-O-O•) [37,38]. The inhibition of the fluorescein breakdown indicates the antioxidant’s protective ability. The assay was carried out as described by Vaario et al. [36], with two technical replicates, by mixing the sample in 0.075 M phosphate buffer pH 7.5 (Merck) with 8.16 × 10^−5^ mM fluorescein and 2,2′-Azobis(2-methylpropionamidine) dihydrochloride (both from Sigma-Aldrich Chemie GmbH, Steinheim, Germany). Trolox (vitamin E analog, (±)-6-Hydroxy-2,5,7,8-tetramethylchromane-2-carboxylic acid) (Sigma-Aldrich Chemie GmbH, Steinheim, Germany) was used as a standard compound and the results are expressed as Trolox equivalents (µmol TE per 100 g).

#### 3.3.3. H_2_O_2_ Scavenging

The hydrogen peroxide (H_2_O_2_) scavenging activity was determined using a method modified from Hazra et al. [39] and Jiang et al. [40]. The assay was carried out according to Vaario et al. [36]. An aliquot of 2 mM H_2_O_2_ (Merck KGaA, Darmstadt, Germany) was added to the reaction mixture with the sample and a mixture containing 2.56 mM ammonium ferrous (II) sulfate (BDH Prolabo) in 0.25 mM H_2_SO_4_ (Merck KGaA) and 27.8 µM xylenol orange disodium salt (Sigma-Aldrich Chemie GmbH, Steinheim, Germany) in 4.4 mM sorbitol (D(-)-sorbitol, AppliChem GmbH). After 30 min incubation, the absorbance of violet-colored ferric-xylenol orange complexes at 560 nm was measured. The assay measures the ability of the sample to scavenge H_2_O_2_ and prevent the oxidation of Fe(II) to Fe(III), which is indicated by the formation of a ferric–xylenol orange complex. The inhibition of the oxidation is expressed as an inhibition % of the reaction and the samples with 100% inhibition activity will remain yellowish. Sodium pyruvate (Sigma-Aldrich) was used as a reference compound.

### 3.4. Antiviral Properties

Adenocarcinomic human alveolar basal epithelial (A549) cells, coxsackievirus B3 (CVB3; Nancy strain, ATCC), and coxsackievirus A9 (CVA9; Griggs strain, ATCC) were used in the assay. CVA9 and CVB3 were produced and purified as previously described [17,41]. The antiviral activity of the round-leaved sundews (field-grown and in vitro propagated) against CVB3 and CVA9 was determined using a cytopathic effect (CPE) inhibition assay, modified from Schmidtke et al. [42]. Briefly, A549 cells were cultured in 96-well flat-bottomed microtiter plates (VWR International) at a density of 12,000 cells/well in DMEM supplemented with 10% FBS, 1% GlutaMAX, and 1% penicillin/streptomycin antibiotics for 24 h at +37 °C. The following day, CVB3 and CVA9 with a virus titer of 2 × 10^5^ PFU/mL and 2 × 10^6^ PFU/mL respectively, were pre-treated with different concentrations of field-grown and lab-grown sundews (10%, 5%, 1%, and 0.1%, *v/v*) for 1 h at +37 °C. The virus-compound mixture was further diluted and added to cells for 48 h incubation in a humidified 5% CO_2_ incubator at +37 °C, to attain MOI of 0.1 and 1 for CVB3 and CVA9, respectively. Virus control (the absence of the compound) and mock infection (the absence of the virus and compound) were used as two controls in the assay. The development of CPE was monitored using light microscopy. After 48 h incubation, cells were washed twice with PBS, before fixing and staining them for 10 min using CPE dye (0.03% crystal violet, 2% ethanol, and 36.5% formaldehyde). Following the staining, cells were washed twice with water and later lysed using a lysis buffer (0.8979 g of sodium citrate and 1M HCl in 47.5% ethanol) to elute the crystal violet. The absorbance of the viable cells was measured spectrophotometrically at 570 nm using the PerkinElmer VICTOR^TM^ X4 multilabel reader. The samples were tested in replicates of nine and the controls in replicates of six. The cytotoxicity of the round-leaved sundews (field-grown and in vitro propagated) at different concentrations (10%, 5%, and 1%, *v/v*) on A549 cells for 24 h was also assessed using CPE Inhibition assay, where a mock infection was used as a control for the experiment.

### 3.5. Stability Analysis by HPLC-DAD

The ethanol extracts of sundew were analyzed with HPLC-DAD (Shimadzu Prominence Liquid Chromatograph system, Shimadzu, Kioto, Japan) with LC-20AP pumps, degasser, autosampler, column oven (+25 °C), and a Prominence Photodiode array SPD-M20A detector (Shimadzu, USA). The HPLC separation was performed in a Waters XBridge C18 reverse-phase column (4.6 × 150 mm, 5 μm) (Waters Corporation, Milford, MA, USA), using (A) H_2_O-phosphoric acid (100:0.1 *v/v*): (B) Methanol (LiChrosolv^®^, Merck KGaA, Germany) gradient elution A:B (0.01 min 65:35; 4.00 min 55:45; 7.00 min 50:50; 14.00 min 50:50; 25.00 min 45:55; 31.00 min 65:35; 32.00 min 65:35 stop) at a flow rate of 0.8 mL/min. The UV absorption of the eluates was monitored using the DAD at 254 nm. The injection volume of the extract solution was 10 μL.

### 3.6. Metabolic Profiling by UPLC-DAD-MS/MS

The qualitative and quantitative UPLC-DAD-ESI-TQ-MS/MS analyses were performed according to Engström et al. [43,44]. The ultrahigh-resolution mass spectrometric analyses were performed using a UPLC-DAD-ESI-QOrbitrap-MS/MS consisting of an Acquity UPLC system (Waters Corp., Milford, MA, USA) coupled with a quadrupole-Orbitrap mass spectrometer (QExactiveTM, Thermo Fisher Scientific GmbH, Bremen, Germany). The column and chromatographic conditions were the same as in the UPLC-DAD-ESI-TQ-MS/MS analyses. Negative and positive ionizations were used in the heated ESI source. In negative ionization, the spray voltage was set at −3.0 kV, sheath gas (N_2_) flow rate at 60, auxiliary gas (N_2_) flow rate at 20, sweep gas flow rate at 0, capillary temperature at +380 °C, S-lens RF level at 60, and in-source collision-induced dissociation (CID) at 30 eV. The parameters were similar in positive ionization, except the spray voltage was set at 3.80 kV. Full-scan MS analyses with a resolution of 140,000 were performed both in the negative and positive ion mode. The mass range of orbitrap was *m/z* 150–2250 and the automatic gain control (AGC) target 3 × 10^6^. MS/MS analyses, namely, dd-MS2 (TopN), were performed in the negative ion mode and the parameters were the following: for full MS, resolution 35,000 and AGC target 3 × 10^6^, and for TopN 3, stepped normalized collision energies 30, 50, and 80 eV, resolution 17,500 and AGC target 1 × 10^5^. The calibration was performed using Pierce ESI Negative Ion Calibration Solution and Pierce ESI Positive Ion Calibration Solution (Thermo Fischer Scientific Inc., Waltham, MA, USA). The data was processed with Thermo Xcalibur Qual Browser software (Version 3.0.63, Thermo Fisher Scientific Inc., Waltham, MA, USA).

### 3.7. Statistical Methods

The differences between the mean values were assessed using a one-way analysis of variance (ANOVA). Statistical analyzes were performed with the IBM SPSS statistics software package (v. 27.0) (IBM, Armonk, NY, USA), except for the antiviral data, which was analyzed using GraphPad PRISM 5 (GraphPad Software, San Diego, CA, USA).

## 4. Conclusions

Round-leaved sundew (*Drosera rotundifolia*) is a small plant, which has been used to treat pulmonary diseases and coughs. It owes its therapeutic properties to secondary metabolites, namely, naphthoquinones and flavonoids. Because of the small size of the plant, it is laborious to obtain the required amounts from natural stands to meet the needs of pharmaceutical, food, and cosmetics industries, which need high amounts of safe and broadly acting bioactive secondary metabolites. Therefore, different cultivation methods, such as in vitro propagation, have been of interest. In this study, we compared the in vitro propagated sundew tissue with the field-grown plant material for their antioxidant and antiviral properties, as well as their metabolic profiles. Field-grown sundew showed significantly higher antioxidant potential whereas the antiviral properties were similar in higher concentrations. Field-grown sundew also contained higher amounts and numbers of potentially effective secondary metabolites in its metabolic profile. We also showed that different drying methods significantly decrease the metabolic composition of the sundew extract and that only freezing could maintain the metabolic profile. Before the in vitro propagated sundew tissue can be considered as an effective alternative to field-grown sundew, the secondary metabolite content and bioactive potential of the in vitro propagated tissue needs to be increased. One possible topic for future studies could be a more significant reduction in the mineral content of the growth media.

## Figures and Tables

**Table 1:** Metabolic profile of the field-grown *Drosera rotundifolia.* Identified based on literature [21,22,23,24,25].

#	RT_TIC_	RT_UV_	Compound	UV λ_max_ (nm) *	Exact Mass Detected	Exact Mass Calculated	Characteristic MS/MS Values	Peak Area **
1	0.59	-	Quinic acid	-	192.06188	192.06339	111, 129, 173	***
2	1.29	1.25	Monogalloyl glucose (β-)	216, 278	332.07366	332.07435	125, 151, 169, 211, 271	601 ± 190
3	2.49	2.46	Digalloyl glycose	226, 273	484.08492	484.08531	125, 151, 169, 211, 271, 331	205 ± 79
4	2.57	2.55	Coumaric acid glycoside	296	326.10021	326.10017	163	164 ± 36
5	3.01	2.98	Methyljuglone diglycoside	232, 298	514.16805	514.16865	188, 351	98 ± 26
6	3.32	3.29	Dihydromyricetin	232, 269, 298	320.05257	320.05322	71, 97, 109, 139, 153, 165, 183	125 ± 38
7	3.39	3.34	Ellagic acid glycoside	253, 361	464.05901	464.05910	172, 216, 244, 284, 301	661 ± 155
8	3.66	3.63	Myricetin-glycoside	208, 225, 257, 357	480.08949	480.09040	179, 271, 316	1154 ± 294
9	3.72	3.67	Hexahydroxyflavone-galloyl-glycoside	200, 226, 265, 298, 359	632.10042	632.10136	109, 137, 151, 179, 317, 479	664 ± 155
10	3.83	3.80	Tetrahydroxyflavone	264	286.04711	286.04774	121, 137,165	118 ± 32
11	3.86	3.84	Unknown	235, 272, 278	348.08403	348.08452	329	27 ± 5
12	3.93	3.90	Ellagic acid	200, 254, 368	302.00557	302.00627	257, 271, 299	4390 ± 1041
13	4.04	4.01	Hyperoside	215, 255, 356	464.09474	464.09548	255, 271, 300, 301	8357 ± 2151
14	4.11	4.08	Galloylhyperoside	226, 264, 257	616.10542	616.10644	151, 301, 463	19,141 ± 4914
15	4.28	4.25	Methyljuglone glycoside	228, 309, 327, 342	352.11540	352.11582	189	1601 ± 353
16	4.33	4.31	Dimethylellagic acid glycoside	246, 370	492.08986	492.09040	270, 298, 313, 328, 476	1271 ± 333
17	4.42	4.39	Kaempferol-galloyl-glycoside	265, 346	600.11073	600.11153	125, 151, 169, 285, 313	349 ± 80
18	4.68	4.64	Quercetin glycoside	254, 357	464.0949	464.09548	107, 151, 179, 255, 271, 300, 301	359 ± 74
19	4.79	4.76	Methylellagic acid	250, 373	316.02181	316.02192	300	152 ± 41
20	4.92	4.89	Hydroxybenzoylhyperin	256, 358	584.11659	584.11661	107, 151, 179, 255, 271, 301, 463	194 ± 46
21	5.17	5.14	Methyljuglone	216, 226, 317, 333, 349,428	190.06185	190.06300	115, 130, 145, 161, 171, 174, 188	-
22	5.25	5.22	Quercetin glycoside gallate	257, 290, 298, 348	616.10603	616.10644	189, 299, 507	464 ± 95
23	5.37	5.34	Quercetin	255, 371	302.04225	302.04266	107, 151, 179	480 ± 120
24	5.70	5.67	Dimethylellagic acid	247, 377	330.03716	330.03757	299, 314	981 ± 249

* The extracts were dilute, which can influence the maxima observed. ** Peak areas were integrated from the photodiode array data at 280 nm and are averages from the three extract replicates; error is calculated from the standard deviations. The peak areas are shown for the peaks with a signal/noise ratio > 2. - means that the signal/noise ratio for the compound was <2 and the peak area has not been determined. *** The compound cannot be detected by photodiode array detection.

**Table 2:** Metabolic profile of the lab-grown propagated *Drosera rotundifolia.* Identified based on literature [21,22,23,24,25].

#	RT_TIC_	RT_UV_	Compound	UV λ_max_ (nm) *	Exact Mass Detected	Exact Mass Calculated	Characteristic MS/MS Values	Peak Area **
1	0.59	-	Quinic acid	-	192.06188	192.06339	-	***
2	1.28	1.24	Monogalloyl glucose (β-)	213, 275	332.07436	332.07435	125, 151, 169, 211, 271	281 ± 9
3	2.57	2.55	Coumaric acid glycoside	295	326.10030	326.10017	163	250 ± 7
4	3.04	3.01	Methyljuglone diglycoside Coumaric acid glycoside isomer	275, 317	514.16817 326.09993	514.16865 326.10017	188, 351 117, 145, 163, 187	146 ± 13
5	3.24	3.21	Two co-eluting unknowns	238, 271	482.10536 370.12584	482.10605 370.12639	57, 125, 151, 179, 193, 283, 463 59, 71, 85, 101, 143, 159, 171, 189, 207	113 ± 2
6	3.41	3.35	Ellagic acid glycoside	253, 361	464.05901	464.05910	132, 145, 172, 216, 244, 284, 301	189 ± 17
7	3.66	3.63	Myricetin-glycoside	255, 305, 311, 349, 356	480.08949	480.09040	124, 151, 179, 271, 287, 316	166 ± 5
8	3.95	3.92	Ellagic acid	254, 369	302.00594	302.00627	257, 271, 299	1305 ± 90
9	4.04	4.01	Hyperoside	253, 357	464.09459	464.09548	107, 151, 179, 255, 271, 300, 301	557 ± 27
10	4.09	4.06 4.08	Galloylhyperoside Hyperoside	255, 349 256, 358	616.1058 5464.09480	616.10644 464.09548	107, 151, 179, 255, 271, 300, 301	158 ± 32
11	4.28	4.25	Methyljuglone glycoside	228, 309, 327, 342	352.11537	352.11582	189	179 ± 132
12	4.35	4.32	Dimethylellagic acid glycoside	246, 370	492.08943	492.09040	270, 298, 313, 328, 476	1435 ± 184
13	4.92	-	Hydroxybenzoylhyperin	-	584.11775	584.11661	151, 179, 215, 243, 271, 287, 301, 316	-
14	5.17	5.14	Methyljuglone	251, 349, 428	190.06183	190.06300	115, 130, 145, 161, 171, 174, 188	-
15	5.24	5.22	Syringetin glycoside	251, 290	508.12154	508.12170	125, 151, 179, 217, 275, 285, 303, 345, 447, 465	37 ± 3
16	5.55	5.52	Spinatoside	257	522.10103	522.10096	185, 229, 257, 285, 300, 313, 328, 343, 491, 506	-
17	5.70	5.67	Dimethylellagic acid	247, 374	330.03703	330.03757	299, 314	379 ± 38

* The extracts were dilute, which can influence the maxima observed. ** Peak areas were integrated from the photodiode array data at 280 nm and are averages from three extract replicates; error is calculated from the standard deviations. The peak areas are shown for the peaks with a signal/noise ratio > 2. - means that the signal/noise ratio for the compound was <2 and the peak area has not been determined. *** The compound cannot be detected by photodiode array detection.

## Data Availability

The data presented in this study are available on request from the corresponding author.

## Sample Availability

The sundew extracts and standards used in the UPLC-DAD-ESI-TQ-MS/MS analyses are not available from the authors.
